# Dental Biofilm–Induced Gingivitis in Children and Adolescents Without Known Systemic Involvement: A Systematic Review

**DOI:** 10.1111/jcpe.70122

**Published:** 2026-03-29

**Authors:** Georgios Tsilingaridis, Nitesh Tewari, Kyriaki Seremidi, William Papaioannou, Rodrigo López

**Affiliations:** ^1^ Division of Paediatric Dentistry, Department of Dental Medicine Karolinska Institute Huddinge Sweden; ^2^ Centre of Paediatric Oral Health Stockholm Sweden; ^3^ Paediatric and Preventive Dentistry, Centre for Dental Education and Research All India Institute of Medical Sciences New Delhi India; ^4^ Department of Paediatric Dentistry, School of Dentistry National and Kapodistrian University of Athens Athens Greece; ^5^ Department of Preventive and Community Dentistry National and Kapodistrian University of Athens Athens Greece; ^6^ Center for Translational Oral Research – Periodontology, Department of Clinical Dentistry University of Bergen Bergen Norway

**Keywords:** adolescent, biofilms, child, dental plaque, gingival diseases, gingivitis

## Abstract

**Aim:**

To synthesize evidence on gingival diseases and conditions in children and adolescents (< 18 years) without known systemic disorder involvement, focusing on their distribution, aetiology, diagnosis, management and oral health–related quality of life (OHRQoL).

**Materials and Methods:**

A systematic review was carried out following PRISMA guidelines, including PubMed Central, Scopus, EMBASE and LILACS, up to January 2025. Clinical trials and observational studies addressing gingival diseases or conditions in healthy individuals under 18 years were included.

**Results:**

The search identified 33,180 studies. Title and abstract screening narrowed these to 2264, of which 433 were full‐text‐reviewed. Ultimately, 269 studies, all restricted to biofilm‐induced gingivitis, were included. Considerable heterogeneity was observed in diagnostic criteria and study quality. Dental biofilm–induced gingivitis was common (52%) and associated with poorer OHRQoL. Key determinants for gingival inflammation included socioeconomic status, oral hygiene behaviours, pubertal changes and malocclusion. Based on current evidence, effective management of gingivitis in children should combine supervised toothbrushing with a fluoridated toothpaste and school‐ or caregiver‐based oral health education. Adjunctive use of chlorhexidine may provide additional benefit in certain clinical situations.

**Conclusion:**

Dental biofilm–induced gingivitis is frequent among children and adolescents and influenced by numerous determinants. Prevention and treatment should emphasise accessible, behaviour‐focused and education‐based strategies for biofilm control.

## 1 Introduction

Periodontal tissues in children and adolescents exhibit anatomical and developmental features that differ markedly from those of adults. These features influence the clinical appearance of gingival inflammation and may modify disease expression during growth and tooth eruption (Bimstein and Matsson 1999).

Gingivitis is a reversible inflammatory reaction of gingival tissues, occurring without periodontal attachment loss, often arising from dental biofilm accumulation on the tooth surface. It results from interactions between the dental biofilm and the host immune–inflammatory reaction (Murakami et al. 2018), as well as local and systemic modifying factors and behavioural influences such as oral hygiene practices (Chapple et al. 2018). It is estimated that everybody experiences gingivitis during their lifetime (Trombelli 2025). In addition to dental biofilm–induced gingivitis, gingival diseases encompass a broad group of conditions with diverse aetiologies, including immune‐mediated, hypersensitivity, infectious, traumatic and neoplastic disorders. These conditions may be localised or may signify underlying systemic involvement, bearing in mind that the absence of evidence of underlying systemic diseases is not proof of its absence (Bell 1897). Although not caused by dental biofilm, their severity may be exacerbated by biofilm‐related inflammation (Chapple et al. 2018). Distinguishing these lesions from dental biofilm–induced gingivitis is critical for accurate diagnosis, appropriate management and timely referral when necessary.

The aim of this systematic review was to address the following focused question: ‘In children and adolescents (< 18 years) without known systemic involvement, what are the main features of these gingival diseases and conditions with respect to occurrence, aetiology, diagnosis, management protocols and impact on oral health–related quality of life (OHRQoL)?’

## 2 Materials and Methods

### 2.1 Protocol Development

The authors were commissioned for conducting this systematic review by the European Federation of Periodontology (EFP) and the European Academy of Paediatric Dentistry (EAPD) for the focused workshop on Gingival and Periodontal Diseases in Children and Adolescents, held in March 2025. The scope was limited to individuals under 18 years of age and the use of clinical categories defined by the 2017 World Workshop on the Classification of Periodontal and Peri‐Implant Diseases and Conditions (Chapple et al. 2018).

The review protocol was based on guidelines including PRISMA, the *Cochrane Handbook*, and ENTREQ (Higgins et al. 2023; Tong et al. 2012), and registered in the Open Science Framework (OSF) (DOI 10.17605/OSF.IO/QCZ54). Studies were selected based on PICOS framework:

- Population: Systemically healthy children and adolescents (< 18 years).
- Phenomenon of Interest: Gingival diseases or conditions not associated with systemic disorders.
- Context: Distribution, aetiology, diagnosis, management and impact on OHRQoL.
- Study design: Clinical trials, cohort, case–control and cross‐sectional studies.

### 2.2 Eligibility Criteria

The review's inclusion and exclusion criteria are presented in Appendix 1.

### 2.3 Search Strategy

A comprehensive literature search was performed across several databases (PubMed Central, Scopus, EMBASE and LILACS) up to January 2025, using a combination of MeSH terms and free‐text keywords (Appendix 2).

### 2.4 Study Selection and Data Extraction

Data from the electronic searches were imported into EndNote (Clarivate USA), and eligibility assessment was done using information from the title, abstract and full‐text analysis in three stages.

Data were extracted and summarised in tables according to outcomes and dentition type. Where feasible, quantitative analyses were conducted on the distribution of the outcomes using random‐effects models, and heterogeneity was assessed with the *I* ^2^ statistics (Higgins et al. 2003) (Appendix 1).

### 2.5 Quality Assessment

Risk of bias was evaluated using tools matched to study design. Randomised trials were appraised with RoB 2.0, focusing on bias related to randomisation, deviations from intended interventions, missing data, outcome assessment and selective reporting. Non‐randomised studies were assessed with ROBINS‐I to address confounding, selection processes, intervention classification and reporting bias. For observational designs, the appropriate JBI checklists were applied (RoB 2.0, ROBINS‐I and JBI checklists) (Moola et al. 2017; Sterne et al. 2016; Sterne et al. 2019; Wells et al. 2014).

The overall strength of evidence was judged using risk of bias assessments and key elements of the GRADE approach (Guyatt et al. 2011). A post hoc decision was made to exclude studies evaluated as having a high risk of bias.

## 3 Results

**FIGURE 1:**
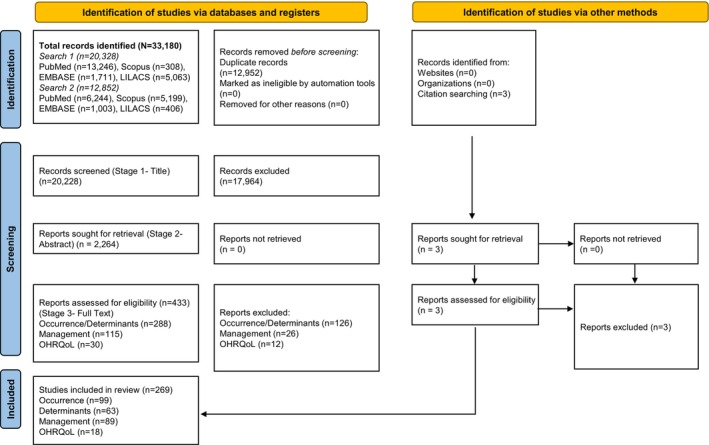
PRISMA 2020 flow diagram for systematic reviews. *Source*: Page et al. (2021). For more information, visit: http://www.prisma‐statement.org/.

In total, 33,180 records were identified, and after removal of duplicates, 20,228 unique records remained for screening. Title screening reduced the pool to 2264 records. Abstract screening resulted in 433 records identified for full‐text evaluation, which finally gave 269 studies fulfilling the inclusion criteria (Figure 1).

Although gingival diseases encompass a wide range of gingival conditions, no studies meeting the inclusion criteria were identified for other than dental biofilm–induced gingivitis.

A total of 99 publications provided information on the occurrence of gingivitis, 63 on determinants, 89 on management and 18 on OHRQoL. No studies were found on the diagnosis of gingivitis.

### 3.1 Occurrence

Studies from 47 countries reported information on the occurrence of gingivitis. The sampling frame for most studies was school‐based. Heterogeneity in the diagnostic criteria, case definitions, sampling strategies and risk of bias was substantial and hindered comparisons of estimates across studies.

**FIGURE 2:**
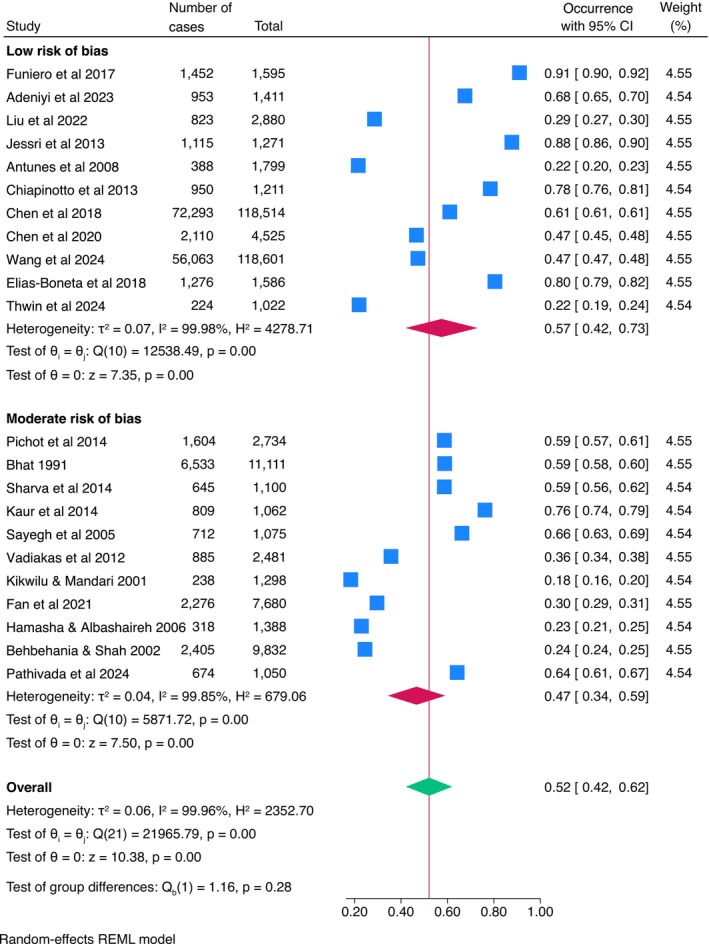
Forest plot of studies reporting the occurrence of gingivitis according to their risk of bias.

**FIGURE 3:**
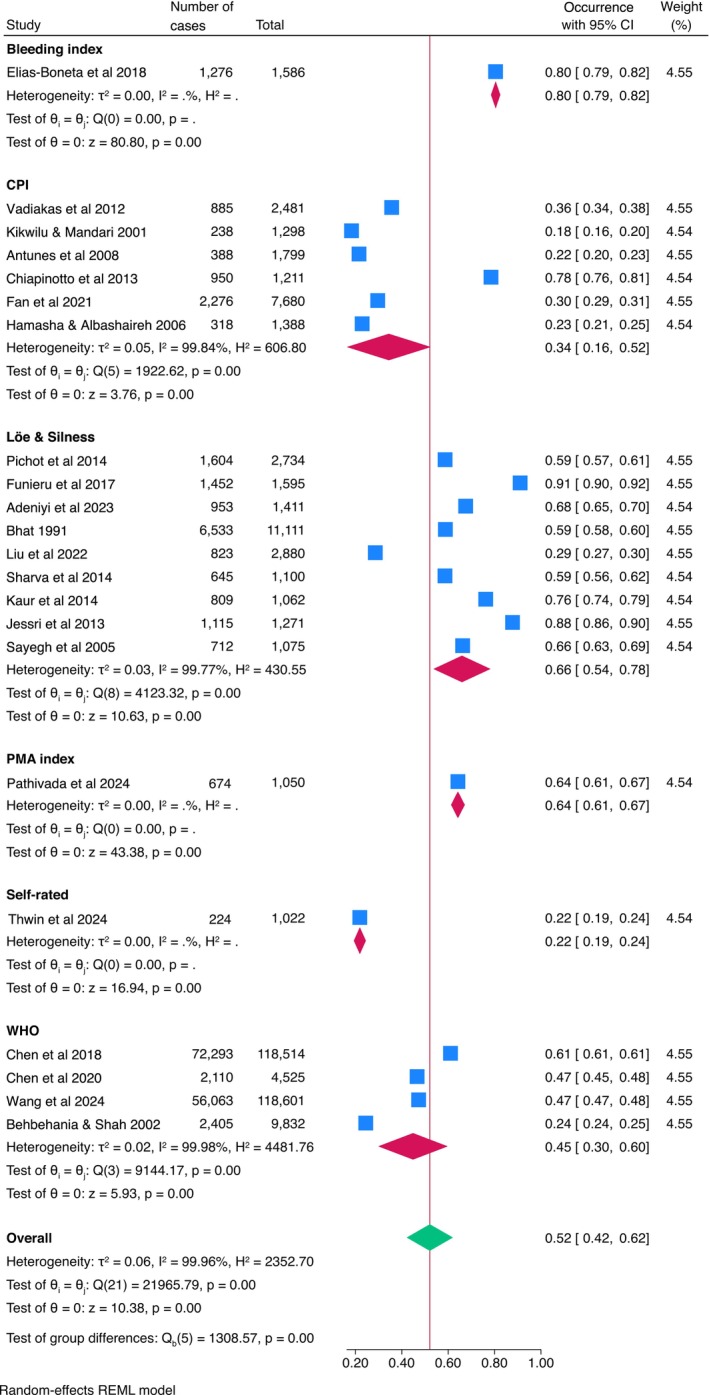
Forest plot of studies reporting the occurrence of gingivitis according to the method used to assess gingivitis.

The most frequently used methods were the Gingival Index (GI) by Loe and Silness (1963) and the Community Periodontal Index (CPI) (World Health Organisation 2013). As numerous definitions of gingivitis were used, when available, bleeding was used as the outcome. Considering that an estimated sample size of 1068 can be calculated assuming an expected 50% gingivitis prevalence and a 95% confidence interval (CI) accuracy within 3% points, it was decided to exclude studies with < 1000 participants and studies evaluated as high risk of bias. A total of 64 studies had < 1000 participants, and 38 studies were evaluated as being of high risk of bias, leaving 22 studies with information on the occurrence of gingivitis. We decided to illustrate variation originating from the methods used and bias with meta‐analysis for occurrence using the ‘meta’ command and ‘esize (proportion)’ option in Stata 19 (Figures 2, 3).

The pooled occurrence estimate for gingivitis was 52% (95% CI: 42%–62%), with substantial heterogeneity (*I* ^2^ > 99%). Estimates differed by the diagnostic method used and threshold, ranging from 34% (CPI‐based studies) to 80% (Bleeding index). The largest included study (*n* = 118,601) reported a 47% prevalence using WHO criteria among 12–15‐year‐old Chinese schoolchildren (Wang et al. 2024). None of the studies used gingivitis criteria defined by the 2017 World Workshop on the Classification of Periodontal and Peri‐Implant Diseases and Conditions (Chapple et al. 2018).

### 3.2 Determinants

Of the 63 studies reviewed, 61 were cross‐sectional, and because the temporality of effects could not be ascertained, they only provided information on associations. A total of 10 articles were excluded because of high risk of bias (Table 1).

**TABLE 1:** Determinants positively associated with gingival inflammation in cross‐sectional studies.

Socioeconomic parameters	
Age	Bashirian et al. (2018),^a^ Fan et al. (2021),^b^ Knack et al. (2019),^a^ Liu et al. (2022), Pathivada et al. (2024),^b^ Wang et al. (2024),^a^ Agbaje et al. (2016),^a^ Jessri et al. (2013),^a^ Lawal and Dosumu (2021)^b^
Gender	Bashirian et al. (2018),^a^ Cortellazzi et al. (2008),^a^ Dummer et al. (1987),^b^ Fan et al. (2021),^b^ Knack et al. (2019),^a^ Wolf et al. (1996),^b^ Thwin et al. (2024),^a^ Simangwa et al. (2018),^b^ Wang et al. (2024),^a^ Agbaje et al. (2016),^a^ Jessri et al. (2013),^a^ Jurgensen and Petersen (2009),^a^ Lawal and Dosumu (2021),^b^ Elias‐Boneta et al. (2018),^a^ Antunes et al. (2008)^a^
Ethnicity	Chiapinotto et al. (2013),^a^ Knack et al. (2019),^a^ Tomazoni et al. (2017),^a^ Simangwa et al. (2018),^b^ Wang et al. (2024),^a^ Antunes et al. (2008)^a^
Education	Knack et al. (2019),^a^ Koga et al. (2020),^a^ Ravera et al. (2012),^a^ Nicolau et al. (2003),^a^ Tomazoni et al. (2017),^a^ Simangwa et al. (2018),^b^ Wang et al. (2024),^a^ Olczak‐Kowalczyk et al. (2024),^a^ Amarasena and Ekanayake (2010),^a^ Medina‐Vega et al. (2024)^a^
Occupation	Bashirian et al. (2018),^a^ Dummer et al. (1987),^b^ Tomazoni et al. (2017),^a^ Kyaw Myint et al. (2020),^a^ Jurgensen and Petersen (2009),^a^ Amarasena and Ekanayake (2010),^a^ Lawal and Dosumu (2021)^b^
Income	Anagnou‐Vareltzides et al. (1983),^a^ Cortellazzi et al. (2008),^a^ Kyaw Myint et al. (2020),^a^ Simangwa et al. (2018),^b^ Agbaje et al. (2016),^a^ Folayan et al. (2022),^a^ Jurgensen and Petersen (2009),^a^ Elias‐Boneta et al. (2018),^a^ Tomazoni et al. (2017),^a^ Nicolau et al. (2003)^a^
Area of residence	Bashirian et al. (2018),^a^ Knack et al. (2019),^a^ Koga et al. (2020),^a^ Ravera et al. (2012),^a^ Tomazoni et al. (2017),^a^ Frencken et al. (1986),^b^ Thwin et al. (2024),^a^ Giacaman et al. (2018),^b^ Jurgensen and Petersen (2009),^a^ Kaur et al. (2014),^b^ Narang et al. (2016),^b^ Vadiakas et al. (2012),^b^ Wang et al. (2024)^a^
Number of goods	Koga et al. (2020)^a^
Household residents	Knack et al. (2019),^a^ Koga et al. (2020),^a^ Tomazoni et al. (2017),^a^ Antunes et al. (2008)^a^
House material	Nicolau et al. (2003)^a^
Family structure	Nicolau et al. (2003)^a^
Immigrant status	Sivakumar et al. (2016)^b^
Private schools	Nagarajappa et al. (2012)^b^
Public school	Elias‐Boneta et al. (2018)^a^
Oral health behaviours	
Brushing frequency	Kyaw Myint et al. (2020),^a^ Liu et al. (2022), Bjarnason et al. (1997),^b^ Wang et al. (2024),^a^ Olczak‐Kowalczyk et al. (2024),^a^ Amarasena and Ekanayake (2010),^a^ Berenie et al. (1973),^b^ Gao et al. (2014),^a^ Jessri et al. (2013),^a^ Kumari et al. (2021),^b^ Sharva et al. (2014),^b^ Smadi (2017)^c^, Weissenbach et al. (1995),^b^ Vadiakas et al. (2012),^b^ Nagarajappa et al. (2012)^b^
Supervised brushing	Olczak‐Kowalczyk et al. (2024)^a^
Brushing time	Liu et al. (2022)
Brushing technique	Lavstedt et al. (1982)^b^
Fluoridated toothpaste	Folayan et al. (2022)^a^
Use of dental floss	Liu et al. (2022), Jessri et al. (2013)^a^
Dietary habits	Kyaw Myint et al. (2020),^a^ Liu et al. (2022), Folayan et al. (2021),^a^ El Tantawi et al. (2021)^a^
Frequency of visits	Fan et al. (2021),^b^ Olczak‐Kowalczyk et al. (2024),^a^ Liu et al. (2022)
No previous visits	Jessri et al. (2013)^a^
Oral health knowledge	
Perception of oral health	Koga et al. (2020),^a^ Tomazoni et al. (2017)^a^
Knowledge	Ravera et al. (2012),^a^ Liu et al. (2022), Gao et al. (2014),^a^ Fan et al. (2021)^b^
Microorganisms	
Bacterial levels	Kyaw Myint et al. (2020)^a^
*Streptococcus mutans*	Weissenbach et al. (1995)^b^
*Actinomyces* spp.	Mitova et al. (2019),^b^ Morinushi et al. (2000),^b^ Pomes et al. (2000)^b^
*Porphyromonas* spp.	Mitova et al. (2019),^b^ Morinushi et al. (2000),^b^ Tankova et al. (2024)^a^
*Treponema* spp.	Mitova et al. (2019),^b^ Tankova et al. (2024)^a^
*Tannerella* spp.	Mitova et al. (2019),^b^ Tankova et al. (2024)^a^
*Prevotella* spp.	Tankova et al. (2024)^a^
*Fusobacterium* spp.	Mitova et al. (2019),^b^ Tankova et al. (2024)^a^
Maloclussions	
Crowding	Cortellazzi et al. (2008),^a^ Feldens et al. (2006),^a^ Tomazoni et al. (2017),^a^ Liu et al. (2022), Antunes et al. (2008)^a^
Overjet	Liu et al. (2022), Kolawole and Folayan (2019)^a^
Anterior open bite	Kolawole and Folayan (2019)^a^
Cross bite	Antunes et al. (2008)^a^
Orthodontic anomalies	Kukletova et al. (2012)^b^, Liu et al. (2022), Medina‐Vega et al. (2024)^a^

^a^ Low risk of bias.

^b^ Moderate risk of bias.

#### 3.2.1 Socioeconomic Variables

One cohort study found that adolescents from lower socioeconomic backgrounds had more gingival bleeding at a 2‐year follow‐up (Sfreddo et al. 2019).

Lower education in adolescents (Knack et al. 2019), lower parental education (Amarasena and Ekanayake 2010; Koga et al. 2020; Medina‐Vega et al. 2024; Nicolau et al. 2003; Olczak‐Kowalczyk et al. 2024; Ravera et al. 2012; Simangwa et al. 2018; Tomazoni et al. 2017; Wang et al. 2024) and attending public schools (Elias‐Boneta et al. 2018; Nagarajappa et al. 2012) were positively associated with gingivitis.

Several studies found lower bleeding scores among children of professionals (white‐collar workers) (e.g., physicians, lawyers, academics), compared to those of skilled, partly skilled and unskilled workers (blue‐collar workers) (Anagnou‐Vareltzides et al. 1983; Dummer et al. 1987; Lawal and Dosumu 2021). However, some studies found no association (Amarasena and Ekanayake 2010; Bashirian et al. 2018; Kyaw Myint et al. 2020). Low family income was positively associated with gingivitis in some studies (Cortellazzi et al. 2008; Knack et al. 2019; Koga et al. 2020), while in others it was not (Medina‐Vega et al. 2024; Nicolau et al. 2003; Tomazoni et al. 2017).

#### 3.2.2 Age

Increasing occurrence of gingivitis is associated with increasing age (Agbaje et al. 2016; Bashirian et al. 2018; Fan et al. 2021; Jessri et al. 2013; Knack et al. 2019; Lawal and Dosumu 2021; Liu, Xu, et al. 2022; Pathivada et al. 2024; Wang et al. 2024).

#### 3.2.3 Gender

In most studies, boys presented more frequently with gingivitis than girls (Agbaje et al. 2016; Antunes et al. 2008; Bashirian et al. 2018; Cortellazzi et al. 2008; Dummer et al. 1987; Jurgensen and Petersen 2009; Lawal and Dosumu 2021; Simangwa et al. 2018; Thwin et al. 2024; Wang et al. 2024). Three studies found no gender differences (Fan et al. 2021; Liu, Xu, et al. 2022; Wolf et al. 1996), whereas one found higher occurrence among girls (Jessri et al. 2013).

#### 3.2.4 Immigrant Status and Ethnicity

Immigrant children and children from non‐White or minority groups had significantly more gingival inflammation, independently of other determinants (Antunes et al. 2008; Jurgensen and Petersen 2009; Knack et al. 2019; Simangwa et al. 2018; Sivakumar et al. 2016; Wang et al. 2024). In one study, the association with ethnicity was not significant (Tomazoni et al. 2017). One study found ‘lighter black‐skinned’ children presented less gingivitis than ‘White’ children (Chiapinotto et al. 2013).

#### 3.2.5 Oral Health Behaviour

Brushing teeth at least twice daily and supervised toothbrushing were significantly associated with improved gingival health (Amarasena and Ekanayake 2010; Berenie et al. 1973; Bjarnason et al. 1997; Gao et al. 2014; Jessri et al. 2011; Kumari et al. 2021; Kyaw Myint et al. 2020; Liu, Xu, et al. 2022; Nagarajappa et al. 2012; Olczak‐Kowalczyk et al. 2024; Pourhashem et al. 2007; Sharva et al. 2014; Smadi 2017; Vadiakas et al. 2012; Wang et al. 2024; Weissenbach et al. 1995).

A cross‐sectional study compared the modified Bass technique with vertical, roll, horizontal and mixed brushing and found that, while higher brushing frequency predicted less plaque, the modified Bass technique was associated with slightly more gingival inflammation despite similar plaque levels, possibly due to a traumatic effect of bristle activity in the gingival sulcus (Lavstedt et al. 1982). Children who seldom or never used fluoride toothpaste had significantly higher odds for developing moderate to severe gingivitis (Folayan et al. 2022). Children who reported infrequent flossing suffered from gingivitis more frequently than children who reported frequent flossing (Jessri et al. 2013; Liu, Xu, et al. 2022).

Lack of regular annual dental check‐ups (once per year) (Fan et al. 2021; Liu, Xu, et al. 2022; Olczak‐Kowalczyk et al. 2024) or never visiting the dentist (Jessri et al. 2011; Liu, Xu, et al. 2022; Wang et al. 2024) was significantly associated with a higher occurrence of gingivitis.

#### 3.2.6 Oral Health Knowledge and Perception of Oral Health

Lower awareness and oral health knowledge among parents and children were associated with poorer gingival health (Fan et al. 2021; Gao et al. 2014; Koga et al. 2020; Liu, Xu, et al. 2022; Ravera et al. 2012; Tomazoni et al. 2017).

#### 3.2.7 Oral Microbiota

Changes in the composition of the sulcular microbiota related to pubertal maturation (Gusberti et al. 1990) and selected microbial profiles, that is, the relative abundance of some bacterial species (Ashley et al. 1988; Kyaw Myint et al. 2020; Mitova et al. 2019; Morinushi et al. 2000; Nadkarni et al. 2015; Pomes et al. 2000; Tankova et al. 2024; Vega‐Chin et al. 2022; Weissenbach et al. 1995), were significantly associated with gingival inflammation.

### 3.3 Other Determinants

Living conditions (Liu, Xu, et al. 2022; Bashirian et al. 2018; Frencken et al. 1986; Giacaman et al. 2018; Jurgensen and Petersen 2009; Kaur et al. 2014; Narang et al. 2016; Ravera et al. 2012; Thwin et al. 2024; Tomazoni et al. 2017; Vadiakas et al. 2012; Wang et al. 2024; Koga et al. 2020; Simangwa et al. 2018; Antunes et al. 2008; Knack et al. 2019; Tomazoni et al. 2017; Nicolau et al. 2003), diet (El Tantawi et al. 2021; Folayan et al. 2021; Kyaw Myint et al. 2020; Liu, Xu, et al. 2022; Wang et al. 2024) and malocclusions (Antunes et al. 2008; Buckley 1981; Cortellazzi et al. 2008; Feldens et al. 2006; Kolawole and Folayan 2019; Kukletova et al. 2012; Liu, Xu, et al. 2022; Medina‐Vega et al. 2024; Tomazoni et al. 2017) were positively associated with gingivitis. Detailed results can be found in Appendix 3.

### 3.4 Management

Eighty‐nine studies reported information on the management of gingivitis (Tables 2 and 3). Forty‐one articles were excluded because of high risk of bias.

**TABLE 2:** Summary of self‐performed interventions for improving gingival health in paediatric populations: Study characteristics and outcomes.

Authors	Study design	Type of management/settings (self‐performed)	Sample size	Age range (years)	Outcome measure	Intervention	Frequency/timing	Total duration	Treatment study group	Treatment control group	Main findings	Risk of bias
Antonio et al. (2007) (Brazil)	Prospective cohort study	Schools	203	9	IBI	OHE	Every 6 months	24 months	Oral health promotion programme	No control	Oral health education lowered IBI scores at 24‐month follow‐up.	Moderate
Bhardwaj et al. (2013) (India)	Prospective cohort study	Schools	276	12 and 15	GI (Löe and Silness)	OHE		4 months	Oral health education programme	No control	Overall mean gingival score decreased significantly after oral health education.	Moderate
D'Cruz and Aradhya (2013) (India)	Randomly selected schools, double‐blind	Schools	568	13–15	GI (Löe and Silness)	OHE	3 and 6 months	9 months	2 groups: Oral health education programme using PowerPoint lecture with or without toothbrushing demonstration	No programme	Reduction in mean gingival score after 9 months for both experimental groups.	Moderate
Sarayuthpitak et al. (2022) (Thailand)	Quasi‐experimental, cohort	Schools	59	10–12	m‐GI	OHE	50 min programme 2 times per week for 6 weeks	3 months	Smartphone endomicroscope and OHE	Dental education as part of the elementary school health curriculum + dentist checkup once per semester	Significant better gingival health at follow‐up in the intervention group	Moderate
Ozoemena et al. (2022) (Nigeria)	Prospective cohort	Schools	96	6–12	GI (Löe and Silness)	OHE	Students visited weekly for 6 weeks	6 weeks	Oral health education and taught tooth brushing technique	No control	Supervised school tooth brushing programme improved gingival scores	Low
Santhosh et al. (2024) (India)	RCT	School	195	12–15	GI (Löe and Silness)	OHE	Participants received reinforcement of the interventions during the first, fourth, and eighth week.	3 months	20‐min OHE + Gr1. Jigsaw Puzzle‐assisted Visual reinforcement and Gr2. Video demonstration	Conventional OHE focusing on toothbrushing techniques	No significant difference between the three groups.	Moderate
van Palenstein Helderman et al. (1997) (Tanzania)	Randomly assigned schools	Schools	431	9–14	BOP	OHE	During the first 3 months, participating schools were visited once every 2 weeks	3 years	Oral health education	No treatment	A significant difference between intervention and control was found for gingival bleeding after 36 months	Low
Saied‐Moallemi et al. (2009) (Iran)	Randomly selected schools	Schools	457	9	BI	OHE	Class‐work: During 3–4 sessions per class in 1 month, each session lasting for 30–45 min Parental‐aid: This intervention (oral health leflet) was provided via the parents at home without giving any additional instructions on oral health at school	3 months	Class‐work group Parental‐aid group Combined group	No treatment	Parental aid and combined led to most positive changes in bleeding scores and healthy gingiva.	Moderate
Shirahmadi et al. (2024) (Iran)	RCT	Schools	190	11–12	CPI	OHE	The researcher evaluated the forms of students in each class once a week to assess the level of adherence to oral health behaviours among students at home	3 months	Theory‐based educational interventions	No intervention	No significant difference on CPI (bleeding)	Low
Subedi et al. (2021) (Nepal)	Randomly selected schools	Schools	240	12–15	GI (Löe and Silness)	OHE	Reinforcement of OHE was done at the third and sixth months in the experimental group.	6 months	Oral health education	No intervention	Gingival index was improved significantly in experimental group.	Low
Englander (1979) (USA)	Prospective cohort	Schools	444	12–16	PI	OHE	Daily during 2 months	3.5 months	Intensive subject oral health education	No education	Decrease in gingivitis in the intervention group	Moderate
Alnouri et al. (2020) (Syria)	RCT and cross over design	Orphanage	17	8–12	GI (Löe and Silness) and GBI	Mouthwash	5 days application + 12 days washout period	20 days	Aloe vera or CHX	Placebo	Aloe vera and CHX had good GI and GBI reduction	Moderate
Bajaj and Tandon (2011) (India)	Block randomisation	Schools	1431	8–12	GI (Löe and Silness)	Mouthwash	Daily rinsing (10 mL for 2 min) after lunch for 9 months.	9 months	Triphala and CHX 0.1%	Placebo	Both Triphala and CHX showed similar effect on gingival health	Moderate
Bhattacharjee et al. (2015) (India)	RCT	Schools	57	8–12	GI (Löe and Silness)	Mouthwash	Rinse for a minimum of 30 s, twice a day, for 2 weeks	2 weeks	Triphala	CHX	No significant difference in mean gingival index between the two groups	Low
Fahim and Zarnigar (2024) (India)	RCT	School	110	8–15	GI (Löe and Silness) and BOP	Mouthwash	Rinse 15 mL morning and evening for 30 days	120 days	Herbal	CHX 0.2%	The difference in GI scores was not statistically significant	Moderate
Nagpal et al. (2020) (India)	RCT	School	20	12–15	GI (Löe and Silness)	Mouthwash	Rinse (10 mL) twice daily for 21 days	3 weeks	Magnetised water	CHX 0.2%	0.2% CHX mouthwash more effective in reducing GI than magnetised water	Moderate
Sharma et al. (2014) (India)	RCT	School and Home	105	12–15	GI (Löe and Silness)	Mouthwash	Rinse (10 mL) twice daily for 21 days	3 months	Herbal mouthwash	CHX 0.2%	Significant reduction in gingival index was found in both herbal and CHX at 21 days and at 1 and 2 months after discontinuing the mouthwash CHX sustained statistically significant reduction in gingival index at 3 months	Moderate
Singhal et al. (2018) (India)	RCT	School and home	135	12–15	GI (Löe and Silness)	Mouthwash	Rinse (10 mL) twice daily for 21 days	28 days	Honey	CHX 0.2%	Both honey and CHX showed significant reductions in gingival scores, but CHX showed the maximum reduction	Low
Vandana et al. (2017) (India)	RCT	School	108	12–15	GI (Löe and Silness)	Mouthwash	Daily mouthrinsing for 6 months	6 months	Stevia Sodium fluoride CHX	Placebo	Stevia showed maximum reduction on gingival scores followed by Sodium fluoride and CHX	Moderate
Kamath et al. (2020) (India)	RCT	School and home	152	8–14	GI (Löe and Silness)	Mouthwash	Rinse (10 mL) twice daily for 4 weeks	6 weeks	Chlorhexidine, aloe vera, and tea tree oil	Placebo	Significant decrease in GI for all groups without difference between the groups	Moderate
Bhor et al. (2021) (India)	RCT	School	72	14–15	GI (Löe and Silness)	Mouthwash	Rinse (30 mL) twice daily for 3 months	3 months	Triphala	CHX 0.12%	No significant differences between the groups.	Low
Silverman et al. (2004) (USA)	RCT	School	58	4–5	GI (Löe and Silness)	Electrical versus manual toothbrush	Time for brushing: 1 min for Oralgiene powered TB and 2 min for Braun Oral‐B Mickey Mouse electric TB and Oral‐B Rugrats 20 manual TB	6 weeks	Electric TB	Manual TB	No clinically meaningful differences between the TB regarding gingival health.	Moderate
Yousuf et al. (2017) (India)	RCT	School	33	12–15	GI (Löe and Silness)	Probiotics	Powder in 30 mL of water and swish once daily for 3 min, for 3 weeks.	3 weeks	Probiotics	Placebo	A significant reduction in gingival status was recorded up to second week of probiotic ingestion	Moderate
Kavitha et al. (2022) (India)	RCT	Orphanage	60	6–12	GI (Löe and Silness)	Probiotics	Probiotic lozenge and placebo lozenge were given twice per day for 30 days	30 days	Probiotics	Placebo	Significant reduction in GI in probiotic group	Low
Shetty et al. (2017) (India)	Prospective cohort	Home	40	9–12	GI (Löe and Silness)	Toothpaste	Brush once daily for 30 days	30 days	Munident herbal dentifrice	Standard toothpaste	Herbal dentifrice has better GI score compared to standard toothpaste	Moderate
Cagetti et al. (2015) (Italy)	RCT	School	40	8–10	BOP	Toothpaste	Twice a day for 2 min each for a 4‐week period	4 weeks	Toothpaste containing fluoride, triclosan and cetylpyridinium chloride and essential oils	Fluoride	No differences between groups	Low
Rich et al. (1989) (USA)	Randomly assigned flossing methods	School	112	8–9	GI (Löe and Silness)	Flossing	Daily flossing or brushing for 4 weeks	4 weeks	Supervised finger‐floss, looped‐floss, floss holder + TB by classroom teachers	TB only	No significant differences in GI scores between the groups.	Moderate
Saha et al. (2012) (India)	Cross‐sectional	Institutions	297	12–15	GI (Löe and Silness)	Chewing stick		4 months	Traditional chewing stick (miswak)	TB + Toothpaste	Miswak users showed a significantly better mean gingival score compared to toothbrush and toothpaste users	Moderate
van Palenstein Helderman et al. (1992) (Tanzania)	Randomly selected schools and randomly matched subjects	School	62	10–13	BOP	Chewing stick	Weekly brushing sessions for 3 months	3 months	Traditional chewing stick (miswak) and OHE TB + OHE	No intervention	No significant differences between Miswak and TB, both reduced gingival bleeding score after 3 months compared to control group.	Low
Saheer et al. (2019) (India)	RCT	School	48	14–15	GI (Löe and Silness) and BI	Miscellaneous	Chewing gum twice daily for 2 weeks	14 days	Sugar free chewing gum (xylitol and sorbitol) + TB 1/day	No gum + TB 1/day	Sugar‐free gum + TB once a day significantly reduced GI and BI score.	Low
Subburaman et al. (2019) (India)	RCT	School	100	6–10	GBI	Miscellaneous	Brushing 2 times/day for 2 min for 3 months	3 months	Manual Musical TB	Manual TB	Musical TB showed a significant reduction in GBI compared to manual TB	Moderate

Abbreviations: BI = bleeding index; BOP = bleeding on probing; CHX = chlorhexidine; CPI = community periodontal index; GBI = gingival bleeding index; GI = gingival index; IBI = interdental bleeding index; m‐GI = modified gingival index; OHE = oral health education; PBI = papillary bleeding index; PI = periodontal index; RCT = randomised controlled trial; TB = toothbrushing.

**TABLE 3:** Summary of professionally managed interventions for improving gingival health in paediatric populations: Study characteristics and outcomes.

Authors	Study design	Type of management/setting (professional management)	Sample size	Age range (years)	Outcome measure	Intervention	Frequency	Total duration	Treatment study group	Treatment control group	Main findings	Risk of bias
Alves et al. (2018) (Brazil)	Cross‐sectional and Cohort	Primary care units	252	3–5	m‐GI by Löe	OHE	From birth to 5 years of age every 3–6 months.	5 years	Attending a public promotion programme in public oral health	Not attending	Promotion programme in public oral health was effective in preventing gingivitis.	Low
Arafa et al. (2025) (Saudi Arabia)	RCT	Dental setting	160	5–7	GI (Löe and Silness)	OHE		6 months	Picture exchange communication system (PECS) on oral health education	Explanation of tooth brushing and proper oral hygiene measures	Participants in the PECS group showed a significantly lower gingival index at follow‐up.	Low
Leghrouz et al. (2024) (Germany)	RCT	Dental setting	58	3–8	PBI	OHE	Oral hygiene instructions to follow for 28 days.	12 weeks	In the differential learning group, participants received printed instructions on tooth brushing in unlabeled sealed envelopes (each exercise for 3 days, then change to the next exercise)	Standard tooth brushing technique instructions	The test group showed lower PBI and remained low at follow‐up.	Low
Shirmohammadi et al. (2022) (Iran)	RCT	Dental setting	90 (51 3mo follow‐up)	2–6	m‐GI	OHE	Every night for 3 months at 9:00 PM and notification on mother's smartphone to brush their child's teeth After 1 month mothers w ere invited with their child to receive a free TB and tooth paste.	3 months	Smartphone application usage by mothers	Educational pamphlet and verbal explanations	Both groups reduced children's m‐GI. The 3‐month follow‐up revealed a better m‐GI in the intervention group.	Moderate
Telford and Murray (1974) (England)	Prospective cohort	Dental setting	54	9–17	GI (Löe and Silness)	OHE	After 1 and 2 months all children were reexamined. The study group encouraged and remotivated verbally.	3 months	Systematic chairside oral hygiene instruction + prophylaxis	Prophylaxis	The intervention group showed a significant improvement in gingival health compared to the control group.	Low
Spets‐Happonen et al. (1985) (Finland)	RCT cross over design	Dental setting	30	8–12	GBI	Mouthwash	Rinse 1 min (10 mL) at home twice a day for 3 days with each solution. 6 days wash out.	18 days	CHX gluconate 0.05% and sodium fluoride (0.025% F)	CHX gluconate 0.05%, sodium fluoride (0.025% F) and strontium chloride (0.10% Sr)	Addition of strontium to the rinsing solution did not statistically significantly reduce gingival bleeding.	Moderate
Kumar et al. (2024) (India)	RCT	Dental setting and Home	108	12–15	GI (Löe and Silness)	Mouthwash	Rinse 30 s (2.5 mL) twice daily for 3 weeks.	21 days	Herbal CHX 0.2%	Placebo	A significant reduction in mean GI scores for CHX and Herbal compared to placebo. CHX significant reductions in GI scores compared to Herbal.	Low
Nezam et al. (2022) (India)	RCT	Dental setting and Home	24	12–15	GI (Löe and Silness)	Mouthwash	Rinse 30 s (10 mL) twice daily for 3 weeks.	6 weeks	Magnetised water	CHX 0.2%	After 21 days the difference was not statistically significant.	Moderate
Owen (1972) (USA)	Prospective cohort	Dental setting	80	2–6	Modification of Ramfjord's index	Electrical versus manual toothbrush	No instructions. After 30 and 90 days both groups were given new TB.	180 days	Electric TB	Manual TB	No statistically significant difference.	Low
Anamika and Kumar (2024) (India)	RCT	Dental setting	100	6–12	GI (Löe and Silness)	Electrical versus manual toothbrush	Every participant received instruction on appropriate brushing methods and was advised to brush for 2 min twice a day.	4 weeks	Electric TB	Manual TB	Electric TB significant decreases gingivitis compared to manual TB.	Moderate
Davidovich et al. (2024) (Israel)	RCT	Dental setting	100	3–6, 7–10	M‐GI	Electrical versus manual toothbrush	Subjects and parents participant received instruction on appropriate brushing methods and was advised to brush for 2 min twice a day.	4 weeks	Electric TB	Manual TB	Electric TB reduced gingivitis compared to manual TB.	Moderate
Pabel et al. (2018) (Germany)	RCT	Dental setting	54	6–9	PBI	Supervised tooth brushing	TB training for 15 days and was performed for 3 min/day at the school's washrooms supervised by dentist.	63 days	Toothbrushing training consisting of 15 days (3 × 5 days, interval 2 days, 3 min/day) Differential learning	Habitual toothbrushing/control, instruction/demonstration of toothbrushing	At all‐time points, PBI was significantly reduced in the treatment group compared to control group.	Moderate
Burton et al. (2013) (New Zealand)	RCT	School dental clinics	100	5–10	GI (Löe and Silness)	Probiotics	The children sucked two lozenges each day for 3 months, one after brushing in the morning and one at night.	7 months	Probiotic	Placebo	No differences between groups.	Moderate
Zickert et al. (1982) (Sweden)	Randomly divided	Dental setting	260	13–14	Gingival units = % of inflamed sites in relation to total number of sites	Professional cleaning	Professional toothcleaning in combination with OH instructions repeated once a month or every 3 months for 2 years.	2 years	Plaque control programme (1) delivered at different intervals (1 and 3 months) and (2) supplemented with mouthrinse with two different fluoride compounds (NaF and MFP)	No control	In both test groups a significant reduction of GU was demonstrated. No difference between the groups.	Moderate
Poulsen et al. (1976) (Denmark)	Randomly assigned	Dental setting	70	7	GI (Löe and Silness)	Professional cleaning	Professional cleansing of the teeth every second week.	12 months	Professional cleansing	No treatment	Significant difference in GI scores after 12 months.	Low
Bretz et al. (2000) (Brazil)	RCT	Dental setting	110	10–15	GI (Löe and Silness)	Gel/varnish	CHX varnish at baseline, 1 week, 3 months.	6 months	CHX varnish	No intervention	CHX varnish resulted in lower % sites with GI 2 and 3 at 6 months.	Low

Abbreviations: BI = bleeding index; BOP = bleeding on probing; CHX = chlorhexidine; CPI = community periodontal index; GBI = gingival bleeding index; GI = gingival index; IBI = interdental bleeding index; m‐GI = modified gingival index; OHE = oral health education; PBI = papillary bleeding index; PI = periodontal index; RCT = randomised controlled trial; TB = tootbbrushing.

#### 3.4.1 Oral Health Education and Supervised Toothbrushing

School‐ and home‐based oral health education primarily reduced gingival inflammation among children who already presented gingivitis, as shown by decreases in gingival bleeding or index scores (Bhardwaj et al. 2013; D'Cruz and Aradhya 2013; Leghrouz et al. 2024; Subedi et al. 2021; van Palenstein Helderman et al. 1997). Supervised brushing, real‐time coaching and instruction by trained personnel were effective therapeutic strategies (Englander 1979; Saied‐Moallemi et al. 2009; Telford and Murray 1974). Digital tools and parent‐focused programmes (Alves et al. 2018; Arafa et al. 2025; Ozoemena et al. 2022; Sarayuthpitak et al. 2022; Shirmohammadi et al. 2022) led to improvements in gingival health, while game‐based or puzzle‐based interventions showed mixed or non‐significant effects on gingival bleeding (Santhosh et al. 2024; Shirahmadi et al. 2024).

In a study, three brushing strategies were compared: (i) habitual brushing without instruction, (ii) brushing with instruction and demonstration and (iii) differential learning in which children brushed with guided variations and challenges (Pabel et al. 2018). All groups brushed under supervision during school hours. The differential learning group showed a significantly greater reduction in papillary bleeding index (PBI) scores.

#### 3.4.2 Electric Versus Manual Toothbrush

Concerning the superiority of electric versus manual toothbrushes on the reduction of gingivitis scores, the results were split, with two earlier studies finding no clinically relevant differences between the two (Owen 1972; Silverman et al. 2004) whereas two recent studies reporting that electric toothbrushes led to significant reductions in mean gingival index (GI, MGI) scores compared to manual toothbrushes (Anamika and Kumar 2024; Davidovich et al. 2024). Only one study reported using rotating‐oscillating toothbrushes, while the others did not report which technology was used, only mentioning electric toothbrushes.

#### 3.4.3 Mouthwashes

Four studies reported a significant decrease in GI scores after 0.1%–0.2% chlorhexidine (CHX) use, as well as after the use of herbal mouthwashes (Bajaj and Tandon 2011; Bhattacharjee et al. 2015; Bhor et al. 2021; Fahim and Zarnigar 2024). A herbal formulation with *Achyranthes aspera* and *Trachyspermum ammi* was compared with 0.2% CHX, which showed non‐inferiority in gingival improvement (Kumar et al. 2024). Neem and mango extract mouthwashes compared to 0.2% CHX all led to significant reductions in gingival scores after 2 months, but only CHX after 3 months (Sharma et al. 2014).

Honey‐based mouthwashes and 0.2% CHX were assessed, showing significant reduction in gingival scores, although CHX outperformed honey‐based mouthwashes (Singhal et al. 2018). When 0.2% CHX mouthwash was compared with aloe vera, the former was seen to significantly reduce gingival scores (Alnouri et al. 2020) or both aloe vera and CHX showed significant reductions in gingival scores (Kamath et al. 2020). A stevioside mouthwash was compared was 0.2% CHX, sodium fluoride and a placebo. GI scores declined slightly less than CHX and sodium fluoride (Vandana et al. 2017).

CHX‐fluoride with or without strontium showed no significant differences between the groups (Spets‐Happonen et al. 1985). Magnetised water was studied for its effectiveness in reducing GI score in comparison with 0.2% CHX, with one study finding CHX more effective (Nagpal et al. 2020) and another one finding no significant difference between the two (Nezam et al. 2022).

#### 3.4.4 Dental Flossing

In a 4‐week school‐based RCT, adding supervised flossing to daily toothbrushing did not improve gingivitis or plaque outcomes compared with brushing alone (Rich et al. 1989).

#### 3.4.5 Professional Tooth Cleaning

Professional tooth cleaning every 2 weeks for a year showed significant reductions in gingival scores in the intervention group compared to controls (Poulsen et al. 1976). Professional cleaning and oral hygiene instruction in a dentist's office, delivered either monthly or every 3 months, combined with fluoride therapy (sodium fluoride, monofluorophosphate, or placebo; in total six subgroups) was compared, and after 2 years all groups showed significant reductions in gingival inflammation, with no significant differences between monthly and tri‐monthly cleaning schedules (Zickert et al. 1982).

In a study, participants received an application of 10% chlorhexidine varnish adjunct to dental prophylaxis, and the percentage of sites with moderate to severe gingivitis significantly decreased in the varnish group compared to controls who received prophylaxis only.

#### 3.4.6 Miscellaneous

Chewing sticks reduced gingival inflammation (Saha et al. 2012; van Palenstein Helderman et al. 1992), while results on the use of toothpastes with different active ingredients (Bye et al. 1989; Shetty et al. 2017; Cagetti et al. 2015) and the use of probiotics showed split results (Burton et al. 2013; Yousuf et al. 2017; Kavitha et al. 2022). Sugar‐free chewing gum and musical toothbrushes reduced bleeding scores (Saheer et al. 2019; Subburaman et al. 2019). Detailed results can be found in Appendix 3.

### 3.5 OHRQoL

A total of 18 studies (some reported across multiple publications; e.g., Krisdapong et al. 2012a, 2012b; Krisdapong, Prasertsom, Rattanarangsima, and Sheiham 2012; Krisdapong, Prasertsom, Rattanarangsima, Sheiham, and Tsakos 2012) with low to moderate risk of bias assessed the impact of gingivitis on OHRQoL; 16 used cross‐sectional study designs (Table 4). Gingival inflammation was mostly measured with the CPI, GI or GBI (gingival bleeding index). The Child Oral Impacts on Daily Performance (c‐OIDP) was the most frequently used instrument (Berhan Nordin et al. 2019; Duan et al. 2025; Kanungo et al. 2023; Krisdapong et al. 2012a; Lawal and Dosumu 2021; Nurelhuda et al. 2010; Peres et al. 2009; Quadri et al. 2022; Wu et al. 2021), followed by the Child Perceptions Questionnaire (CPQ11–14), Condition‐specific Impacts (CS‐impacts) and Autoquestionnaire Qualite de Vie Enfant Image (AQVEI) (da Silva Pde et al. 2015; Krisdapong et al. 2012a, 2012b; Kumar et al. 2017; Ortiz et al. 2020; Paula et al. 2012; Tomazoni et al. 2014). Most studies found gingival inflammation negatively impacting OHRQoL, although some reported non‐significant associations.

**TABLE 4:** Studies on the association between the gingivitis and oral health–related quality of life.

Authors (country)	Study design	Setting	Sample size	Age range (years)	Outcome measure	OHRQoL tool	Results	Risk of bias
Krisdapong et al. (2012a) (Thailand)	Cross‐sectional	National survey	811	15	CPI	CS‐impact	Moderate/higher CS‐impacts was significantly associated to increasing number of sextants with gingivitis.	Low
Krisdapong et al. (2012b) (Thailand)	Cross‐sectional	National survey	1063	12	CPI	CS‐impact	Moderate/Higher CS‐impacts was significantly associated to increasing number of sextants with calculus with gingivitis but not gingivitis alone in 12‐year‐olds.	Low
Krisdapong, Prasertsom, Rattanarangsima, and Sheiham (2012) (Thailand)	Cross‐sectional	National survey	1874	12 and 15	CPI	C‐OIDP	Gingivitis significantly associated with impacts on relaxing, smiling, study and social contact in 12‐year‐olds but not 15‐year‐olds	Low
Krisdapong, Prasertsom, Rattanarangsima, Sheiham, and Tsakos (2012) (Thailand)	Cross‐sectional	National survey	1874	12 and 15	CPI	CS‐impact	Gingivitis with or without calculus was significantly associated to any level of CS‐impacts in 12‐ and 15‐year‐olds. Moderate/Higher CS‐impacts was significantly associated to increasing number of sextants with gingivitis without calculus in 15‐year‐olds but not 12‐year‐olds.	Low
Berhan Nordin et al. (2019) (Malaysia)	Cross‐sectional	Schools	249	11–12	CPI, GBI	C‐OIDP	No significant association between GBI and C‐OIDP score.	Low
Duan et al. (2025) (China)	Cross‐sectional	Schools	485	12	Bleeding presence or absence	C‐OIDP	No significant association between BOP and C‐OIDP score.	Low
Lawal and Dosumu (2021) (Kenya)	Cross‐sectional	Schools	976	10–14	GBI	C‐OIDP	There was no statistically significant association between clinically evident bleeding and impacts on the quality of life	Low
Quadri et al. (2022) (Saudi Arabia)	Cross‐sectional	Schools	700	12–14	GI Löe and Silness	C‐OIDP	Poorer gingival statuses associated with C‐OIDP greater scores.	Low
Wu et al. (2021) (China)	Cross‐sectional	Schools	89,582	12–15	CPI	C‐OIDP	Number of teeth with gingival bleeding were significantly associated with C‐OIDP scores.	Low
Nurelhuda et al. (2010) (Sudan)	Cross‐sectional	Schools	1117	12	GI Löe and Silness	C‐OIDP	GI score was not significantly associated with C‐OIDP scores	Low
Kanungo et al. (2023) (India)	Cross‐sectional	Schools	1034	12	Bleeding presence or absence	C‐OIDP	Present bleeding was significantly associated to C‐OIDP scores	Low
Kumar et al. (2017) (India)	Cross‐sectional	Schools	1342	11–12	CPI	CPQ 11–14	Gingivitis was not associated to OHRQoL	Low
Ortiz et al. (2020) (Brazil)	Cohort	Schools	1134	12	CPI	CPQ 11–14	Gingivitis was associated with overall score CPQ‐score and emotional well‐being.	Low
Ripardo et al. (2025) (Brazil)	Cross‐sectional	Schools	406	12	CPI	CPQ 11–14	Greater gingival bleeding was directly associated with poor self‐rated oral health. Poor self‐rated oral health was directly associated with poor OHRQoL.	Low
Tomazoni et al. (2014) (Brazil)	Cross‐sectional	Schools	1134	12	CPI	CPQ 11–14	Extensive levels of gingivitis have a higher impact on a child's self‐perceived quality of life than that in children with low‐level/no gingivitis. The association between extensive‐level gingivitis and CPQ11–14 scores found mainly in the emotional well‐being domain	Low
Paula et al. (2012) (Brazil)	Cross‐sectional	Schools	515	12	Bleeding presence or absence	CPQ 11–14	Presence of bleeding showed significant associations with worse OHRQoL.	Low
da Silva Pde et al. (2015) (Brazil)	Cross‐sectional	Schools	64	12	CPI	AQVEI	There was no significant difference between groups with gingivitis versus controls and AUQEI scores.	Moderate
Peres et al. (2009) (Brazil)	Cohort	Home visits	339	12	% teeth with bleeding after 10 s	C‐OIDP	Adolescents presenting severe GB, showed higher OIDP score when compared with those free of GB	Low

Abbreviations: AQVEI = Auto questionnaire Qualite de Vie Enfant Image; C‐OIDP = Child‐Oral Impact in Daily Performance; CPI = community periodontal index; CPQ 11–14 = Child Perceptions Questionnaire; CS‐impact = condition‐specific impact; GBI = gingival bleeding index; GI = gingival index; OHRQoL = oral health–related quality of life.

## 4 Discussion

### 4.1 Occurrence and Determinants

Dental biofilm–induced gingivitis is very common among individuals under 18. However, owing to considerable heterogeneity in the methods used, comparisons of different occurrence estimates across study groups are not possible.

This review highlights that gingivitis in children is influenced by sociodemographic, behavioural and biological determinants. Socioeconomic disadvantage—reflected by lower income, lower parental education, parental occupation, school type, ethnicity and immigration status—was positively associated with gingival inflammation (Antunes et al. 2008; Cortellazzi et al. 2008; Koga et al. 2020; Lawal and Dosumu 2021; Liu, Xu, et al. 2022; Medina‐Vega et al. 2024; Simangwa et al. 2018; Sivakumar et al. 2016; Tomazoni et al. 2017). Behavioural factors, such as toothbrushing with a fluoride‐containing paste twice daily, supervised brushing and regular dental visits, were positively associated with better gingival health (Gao et al. 2014; Jessri et al. 2013; Olczak‐Kowalczyk et al. 2024; Wang et al. 2024). In contrast, low oral health knowledge, poor hygiene routines and inadequate dental care access mediated the effects of low socioeconomic status (Koga et al. 2020). These findings emphasise the need for preventive strategies that target both individual behaviours and caregiver involvement through education and support. Health beliefs and self‐efficacy seem to play a role, often shaped by socioeconomic and cultural backgrounds. This interplay of direct and indirect effects highlights the complexity behind sociodemographic determinants: exerting influence through multiple mechanisms such as hygiene practices, healthcare utilisation and psychosocial environment.

Studies in this review suggest that the occurrence of gingivitis increases with age during childhood and early adolescence (Agbaje et al. 2016; Bashirian et al. 2018; Fan et al. 2021; Jessri et al. 2013; Knack et al. 2019; Lawal and Dosumu 2021; Liu, Wong, et al. 2022; Pathivada et al. 2024; Wang et al. 2024). One reason for this could be changes in the oral microflora related to puberty (Gusberti et al. 1990) and to hormonal changes during puberty, particularly elevated levels of sex steroid hormones, which modulate the host immune–inflammatory response and result in an exaggerated gingival reaction to dental plaque (Murakami et al. 2018). Another explanation can be behavioural changes related to adolescence. Boys experience gingivitis more frequently than girls, maybe due to differences in oral health behaviours (Furuta et al. 2011).

Tobacco smoking is a well‐established cause of periodontitis in adults. However, we did not identify studies assessing the role of direct or indirect exposure to tobacco in gingival diseases and conditions among children or adolescents.

#### 4.1.1 Management

Numerous studies have found that school‐ and caregiver‐based oral health education can reduce gingival inflammation and improve oral hygiene behaviours. School‐based programmes, particularly those incorporating interactive elements like real‐time plaque disclosure (Telford and Murray 1974) or supervised brushing (Englander 1979; Pabel et al. 2018), can improve gingival health. This is consistent with Kim and Kim's meta‐analysis, which found that educational interventions yielded substantial improvement of plaque and gingival indices (Kim and Kim 2024). Several studies highlight that the effectiveness of health education is influenced not only by its content but also by its delivery and surrounding social context (D'Cruz and Aradhya 2013; Saied‐Moallemi et al. 2009). Furthermore, maternal education programmes, continuing from birth through early childhood, have shown significant improvements in children's gingival health, underscoring the benefits of engaging caregivers over time (Alves et al. 2018).

Regarding the effects of electric versus manual toothbrushes on the reduction of gingivitis scores, the observed differences can be explained by serious methodological limitations, including inconsistencies in study design, inadequate reporting of allocation procedures and differences in instruction given to study participants (Anamika and Kumar 2024; Davidovich et al. 2024). Consequently, no conclusion can be drawn on differences between the use of electric or manual toothbrushing and gingivitis among children or adolescents.

Supervised miswak use may help control gingivitis, offering an advantage in populations without access to toothbrushes (Saha et al. 2012; van Palenstein Helderman et al. 1992).

Studies reporting an association between self‐reported dental flossing and lower gingivitis frequency relied on questionnaire data, with the consequent risk of reporting bias (Jessri et al. 2013; Liu, Xu, et al. 2022). Flossing may function as a proxy indicator for overall oral health awareness or higher socioeconomic status, and children who report flossing are also likely to brush more effectively or more frequently. Rich et al. (1989) evaluated flossing under controlled conditions, concluding that toothbrushing alone achieved clinical results comparable to combined toothbrushing and flossing. Evidence suggests that flossing does not significantly reduce gingivitis in children, whereas supervised toothbrushing and regular use of fluoridated toothpaste improve gingival health. Despite professional tooth cleaning every second week or once a month resulted in some improvement of gingival health in children, these interventions were unrealistic and not commensurate with the occurrence of gingivitis (Poulsen et al. 1976; Zickert et al. 1982).

Mouthwashes based on 0.1%–0.2% chlorhexidine (CHX) and herbal formulations were shown to reduce gingival inflammation in children (Bajaj and Tandon 2011; Bhattacharjee et al. 2015; Bhor et al. 2021; Kamath et al. 2020; Singhal et al. 2018). Although honey‐based mouthwashes showed gingival health benefits, their sugar content raises concerns about caries risk (Singhal et al. 2018).

Chlorhexidine varnish applied 2–3 times over 6 months also resulted in a reduction of moderate to severe gingivitis (Bretz et al. 2000). However, considering the favourable effect of trained mechanical plaque control, it is unnecessary to recommend varnishes unless mechanical plaque control is hindered in individual cases.

#### 4.1.2 OHRQoL

While most studies suggest a detrimental impact of gingivitis on OHRQoL among the young, the results are not consistent. Bleeding gingiva may be associated with the fear of blood, which could further hinder toothbrushing (Lawal and Dosumu 2021).

When different components of OHRQoL were considered, significant associations with impacts on relaxing, smiling and social contact were reported (Krisdapong, Prasertsom, Rattanarangsima, and Sheiham 2012). These associations can be attributed to both the subjective nature of the concept and the effect of patient‐related confounding factors. However, it should be noted that they were only evident in younger children, which might indicate that as children get older, they tend to focus on things related to their appearance rather than their oral health. Previous studies have also reported an impact on an eating component (Berhan Nordin et al. 2019; Zahra et al. 2024), related to dental pain caused by bleeding gingiva.

## 5 Conclusions

From this review, we found that gingivitis among children and adolescents without known systemic disorder involvement was common and influenced by a complex interplay of biological, behavioural and sociodemographic determinants. Socioeconomic disadvantage, inadequate oral hygiene practices, limited oral health knowledge, pubertal changes and malocclusions contribute to gingival inflammation. Effective prevention and management strategies should be based on interventions early in life, focusing on education and behavioural changes, particularly those delivered through schools and caregivers. Supervised toothbrushing remains the cornerstone of gingivitis control, with selective adjunctive use of antimicrobial mouth washes showing additional benefit.

## Author Contributions

All authors contributed to the study conception and design, data acquisition, analysis and interpretation and manuscript drafting, and gave their final approval of the version to be published.

## Funding

The authors have nothing to report.

## Conflicts of Interest

The authors declare no conflicts of interest.

## Data Availability

The data that support the findings of this study are available from the corresponding author upon reasonable request.

## Appendix 1

### Materials and Methods

The authors were invited carry out this review by the European Federation of Periodontology (EFP) and the European Academy of Paediatric Dentistry (EAPD) to provide the basis for discussions during the joint EFP/EAPD Focused Workshop on ‘Gingival and periodontal diseases and conditions in children and adolescents’ held in Madrid, Spain, in March 2025. The organising committee limited the scope of the workshop to children and adolescents < 18 years, and the clinical categories included in the Consensus Report of Working Group 1 of the 2017 World Workshop on the Classification of Periodontal and Peri‐Implant Diseases and Conditions (Chapple et al. 2018).

The protocol was registered at the Open Science Framework (OSF) (DOI 10.17605/OSF.IO/QCZ54).

The study was designed according to the Preferred Reporting Items for Systematic Review and Meta‐analysis statement (PRISMA) (Page et al. 2021), the *Cochrane Handbook for Systematic Reviews* (version 6.4., updated August 2023) (Higgins et al. 2023), *En*hancing *T*ransparency in *Re*porting the synthesis of *Q*ualitative research ENTREQ (Tong et al. 2012), and the instructions from the Scientific Committee of the EFP Focused Workshop with the EAPD taking into consideration the following specific items for the PICOS question:

– *P*opulation: Systemically healthy children and adolescents (< 18 years).
– Phenomena of *I*nterest: Gingival diseases or conditions not associated with systemic disorders.
– *C*ontext: Distribution of conditions and risk factors, diagnosis, management protocols and effects on oral health–related quality of life (OHRQoL).
– *S*tudy design: Clinical trials (randomised, non‐randomised, controlled, pre–post studies), cohort studies, case–control studies and cross‐sectional studies.

#### Inclusion Criteria

- Studies including children and adolescents (< 18 years). Studies including older individuals were included if they provided separate relevant information for participants < 18 years.
- Studies include individuals with dental biofilm–induced gingivitis.
- Studies reporting the distribution of conditions, aetiology, diagnosis, management protocols and/or OHRQoL.
- Study design: Clinical trials (randomised, non‐randomised controlled, pre–post studies), cohort studies, case–control studies and cross‐sectional studies.

#### Exclusion Criteria

- Studies not published in English.
- Pre‐clinical in vitro or animal studies.
- Studies with high risk of bias (assessed using a validated tool).
- Systematic and narrative reviews, editorials.
- Studies reporting on conditions affecting the periodontal attachment.
- Studies that include individuals with known systemic diseases/conditions that are associated with periodontal manifestations.

A minimum sample size criterion was applied only to studies assessing occurrence, as specified in the Occurrence section. No restrictions were imposed regarding the duration of follow‐up.

A comprehensive search strategy was developed following discussion between members of the research team and conducted electronically in the PubMed Central, Scopus, EMBASE and LILACS databases for studies up to and including September 2024. The search was updated on 1 January 2025. The search strategy used a combination of MeSH terms and free text words (see Appendix 2).

##### Risk of Bias

For RCTs or controlled clinical trials (CCTs), the quality assessment followed the Cochrane RoB 2.0 tool (Sterne et al. 2019) and the ROBINS‐I (Risk Of Bias In Non‐randomized Studies – of Interventions) tool (Sterne et al. 2016), respectively. Observational studies were assessed with Joanna Briggs Institute's Critical Appraisal Checklist (JBI) (Moola et al. 2017; Wells et al. 2014).

##### Study Selection

Titles and abstracts from the electronic searches were imported into EndNote online (Clarivate USA), and removal of duplicate studies was performed electronically. Eligibility assessment was done using information from the title, abstract and full‐text analysis in three stages.

At the first stage, three reviewers (N.T., K.S., G.T.) independently screened titles and excluded those irrelevant to the review. At a second stage, abstracts were screened independently by the same reviewers for inclusion in the review.

During this stage, focus was on the contexts of the review, namely distribution of the condition, aetiology, diagnosis, management protocols and OHRQoL. Two reviewers each screened the records as per the four contexts: N.T., R.L. for distribution of conditions; G.T., K.S. for aetiology; G.T., R.L. for diagnosis; R.L., W.P. for management protocols; and N.T., K.S. for OHRQoL. In the third stage, the full‐text versions of candidate studies for each of the contexts were independently assessed by the same reviewers. In case of disagreement, consensus was established by discussion.

##### Data Extraction

A discussion was performed to establish the data extraction form for each of the contexts, and they were pilot‐tested in a sample of five studies for each context. The full‐text articles included in the above stage were discussed among the authors to achieve consensus. For the studies related to management protocols, the data on reported outcomes were extracted at baseline and the longest follow‐up visit (either the exact value in each visit or the change between visits).

##### Summary Measures and Synthesis of Results

The extracted data were analysed and summarised in tables. Results were reported based on

- Exposure: Dental biofilm–induced gingivitis;
- Outcomes: Distribution of conditions, determinants, diagnosis, management protocols and OHRQoL;
- Type of dentition: Deciduous, mixed, permanent.

Descriptive or qualitative summary conclusions were drawn for the occurrence, determinants, management and the effect on OHRQoL.

Quantitative analyses were conducted for data on the distribution of dental biofilm–induced gingivitis using random‐effects models and assessing the heterogeneity across studies using the *I* ^2^ values (Higgins et al. 2003). Statistical significance was defined as a *p*‐value < 0.05.

##### Strength of the Evidence

The evidence was appraised using the risk of bias and an approach based on other elements of GRADE (Grading of Recommendations, Assessment, Development and Evaluation) (Guyatt et al. 2011). A post hoc decision was made to exclude the studies evaluated as with high risk of bias. The usual protocol was to perform screening, then data extraction, and later risk of bias analysis. These steps were followed for this systematic review as well, but unlike the usual systematic reviews, this work is an invited review to develop a consensus statement. Therefore, the paper was not focused on one part but included multiple research questions. The aspects of occurrence and determinants, along with OHRQoL, were derived from observational studies with high heterogeneity. This is a common problem seen in such study designs, and especially in the field such as gingival diseases of children, where several outcome assessment methods have been used by researchers. Similarly, the management of gingival diseases involved interventional studies and required recommendations that could have better generalisability and direction. This was deemed to be achieved by including only the studies that had a moderate or low risk of bias.

## Appendix 2

### Information Sources and Search

A comprehensive search strategy was developed following discussion between members of the research team and conducted electronically in PubMed Central, Scopus, EMBASE, Web of Sciences and LILACS databases for studies up to and including September 2024. The search was updated on 1 January 2025. The search strategy used a combination of MeSH terms and free text words.

#### Search 1—[Exposure (Gum Diseases) AND Population (Children and Adolescents)]

Field 1: Gingivitis OR Gingivitides OR Gingival‐Pocket OR Dental‐plaque‐induced‐gingivitis

Field 2: Child OR Children OR Adolescents OR Adolescence OR Youth OR Adolescent OR Youths OR Teen OR Teens OR Teenager OR Teenagers OR Young‐adults

#1 AND #2

#### Search 2—[Exposure (Risk Factors) AND Gums AND Population (Children and Adolescents)]

Field 1: Dental‐Plaque OR Smoking OR Tobacco‐Smoking OR Vape OR Vaping OR E‐cigarette OR Smokeless‐Tobacco OR Menstrual‐cycle OR Oral‐contraceptives OR Bacteria OR Bacterial OR Neisseria‐gonorrhoeae OR Treponema‐pallidum OR Mycobacterium‐tuberculosis OR Streptococcal OR Viral OR Virus OR Coxsackie‐virus OR hand‐foot‐and‐mouth‐disease OR Herpes‐simplex‐Virus‐I OR HSV‐I OR Herpes‐simplex‐Virus‐II OR HSV‐II OR Herpes‐simplex‐I OR Herpes‐simplex‐II OR Varicella‐zoster OR chicken‐pox OR shingles OR Molluscum‐contagiosum OR Human‐papilloma‐virus OR squamous‐cell‐papilloma OR condyloma‐acuminatum OR verruca‐vulgaris OR Fungal OR Fungus OR Candidosis OR mycoses OR histoplasmosis OR aspergillosis OR Hypersensitivity OR Contact‐allergy OR Erythema‐multiforme OR Physical‐Trauma OR mechanical‐trauma OR Frictional‐keratosis OR Factitious‐injury OR Self‐injury OR self‐harm OR Chemical‐burn OR toxic‐burn OR Thermal‐insults OR Burns OR Amalgam‐tattoo OR Puberty OR pubertal OR Pre‐puberty OR Pre‐pubertal OR Pregnancy

Field 2: Gum OR Gums OR Gingiva OR Gingival OR Interdental‐Papilla OR Papilla‐Interdental

Field 3: Child OR Children OR Adolescents OR Adolescence OR Youth OR Adolescent

OR Youths OR Teen OR Teens OR Teenager OR Teenagers OR Young‐adults

#1 AND #2 AND #3

The reference lists of the included studies were also hand searched.

## Appendix 3

### Extended Results on Determinants and Management Not Presented in Detail in the Main Manuscript

#### Determinants

##### Living Conditions

In all but one study (Liu, Xu, et al. 2022), rural or semi‐urban residence was positively associated with gingival bleeding (Bashirian et al. 2018; Frencken et al. 1986; Giacaman et al. 2018; Jurgensen and Petersen 2009; Kaur et al. 2014; Narang et al. 2016; Ravera et al. 2012; Thwin et al. 2024; Tomazoni et al. 2017; Vadiakas et al. 2012; Wang et al. 2024). Lower material wealth (Koga et al. 2020; Simangwa et al. 2018), household crowding (Antunes et al. 2008; Knack et al. 2019; Koga et al. 2020; Tomazoni et al. 2017), non‐brick housing at birth, and adolescents from reconstituted families (i.e., households with a step‐parent) (Nicolau et al. 2003) were significantly associated with increased gingival bleeding.

##### Diet

Frequent consumption of refined carbohydrates between meals was significantly associated with an increased risk of moderate to severe gingivitis (El Tantawi et al. 2021; Folayan et al. 2021; Kyaw Myint et al. 2020; Liu, Xu, et al. 2022; Wang et al. 2024).

##### Malocclusions

Crowding and overjet were associated with gingival inflammation (Antunes et al. 2008; Buckley 1981; Cortellazzi et al. 2008; Feldens et al. 2006; Kolawole and Folayan 2019; Kukletova et al. 2012; Liu, Xu, et al. 2022; Medina‐Vega et al. 2024; Tomazoni et al. 2017).

#### Management

##### Chewing Stick (Miswak)

Two studies documented favourable effects of the use of miswak, a traditional chewing stick derived from the plant *Salvadora persica*, on gingival inflammation (Saha et al. 2012; van Palenstein Helderman et al. 1992).

##### Toothpastes

Two dentifrices containing Microsil, a proprietary antimicrobial silica compound, compared to a placebo paste, were evaluated and one Microsil formulation showed statistically significant reductions in gingivitis over time (Bye et al. 1989). Fluoride toothpaste was compared to Munident, an Ayurvedic herbal dentifrice and found no differences (Shetty et al. 2017). In a 4‐week double‐blind RCT in schoolchildren, a toothpaste containing triclosan, cetylpyridinium chloride and essential oils produced greater plaque reduction than a standard fluoridated toothpaste, with no difference in bleeding on probing (Cagetti et al. 2015).

##### Probiotics

The use of the probiotic strain *Streptococcus salivarius* M18 in lozenge form showed no statistically significant changes in gingival scores compared to a control group (Burton et al. 2013).

Powder formulations, containing *Lactobacillus acidophilus* , *Bifidobacterium longum* , Bifidobacterium bifidum and Bifidobacterium lactis or *lactic acid bacillus*, administered daily as mouth rinse for 3 weeks showed statistically significant improvements in gingival scores compared to baseline and placebo (Yousuf et al. 2017). In another study, the probiotic group received lozenges containing *Streptococcus faecalis* , *Clostridium butyricum* , *Bacillus mesentericus* and *Lactobacillus sporogenes* twice daily for 1 month and showed significant reductions in gingival scores compared to the placebo group (Kavitha et al. 2022).

##### Miscellaneous

The use of sugar‐free chewing gum sweetened with xylitol or sorbitol twice daily after meals showed significant reductions in gingival and bleeding scores compared to controls (Saheer et al. 2019).

The use of a musical toothbrush showed significantly better outcomes in the Gingival Bleeding Index compared to a regular toothbrush (Subburaman et al. 2019).
